# Revealing the hidden RBP–RNA interactions with RNA modification enzyme‐based strategies

**DOI:** 10.1002/wrna.1863

**Published:** 2024-06-22

**Authors:** Hua Jin, Chong Li, Yunxiao Jia, Yuxuan Qi, Weilan Piao

**Affiliations:** ^1^ Laboratory of Genetics and Disorders, Key Laboratory of Molecular Medicine and Biotherapy Aerospace Center Hospital, School of Life Science, Beijing Institute of Technology Beijing People's Republic of China; ^2^ Advanced Technology Research Institute, Beijing Institute of Technology Jinan People's Republic of China; ^3^ Faculty of Science University of British Columbia Vancouver British Columbia Canada

**Keywords:** RNA modification, RNA tagging, RNA‐binding protein, STAMP, TRIBE

## Abstract

RNA‐binding proteins (RBPs) are powerful and versatile regulators in living creatures, playing fundamental roles in organismal development, metabolism, and various diseases by the regulation of gene expression at multiple levels. The requirements of deep research on RBP function have promoted the rapid development of RBP–RNA interplay detection methods. Recently, the detection method of fusing RNA modification enzymes (RME) with RBP of interest has become a hot topic. Here, we reviewed RNA modification enzymes in adenosine deaminases that act on RNA (ADAR), terminal nucleotidyl transferase (TENT), and activation‐induced cytosine deaminase/ApoB mRNA editing enzyme catalytic polypeptide‐like (AID/APOBEC) protein family, regarding the biological function, biochemical activity, and substrate specificity originated from enzyme selves, their domains and partner proteins. In addition, we discussed the RME activity screening system, and the RME mutations with engineered enzyme activity. Furthermore, we provided a systematic overview of the basic principles, advantages, disadvantages, and applications of the RME‐based and cross‐linking and immunopurification (CLIP)‐based RBP target profiling strategies, including targets of RNA‐binding proteins identified by editing (TRIBE), RNA tagging, surveying targets by APOBEC‐mediated profiling (STAMP), CLIP‐seq, and their derivative technology.

This article is categorized under:

RNA Interactions with Proteins and Other Molecules > Protein‐RNA Recognition
RNA Processing > RNA Editing and Modification

## Graphical abstract

The timeline for development of RNA‐binding proteins' target RNA profiling methods.

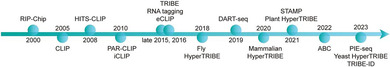

## 1 INTRODUCTION

RNA‐binding proteins (RBPs) interact with RNA inside cells, and ensure the precisely‐regulated RNA life cycle such as co‐transcriptional processing, nuclear export, functioning, and degradation (Gerstberger et al., 2014). Considering that RBPs are expected to account for over 10% of human genes (Mukherjee et al., 2019), the protein–RNA interaction forms an extremely intricate network within the cell and underlies fundamental cellular activity (Darnell, 2010; Fecko et al., 2007; McHugh et al., 2014; Wang, Tidei, et al., 2015).

Defects in RBPs affect various biological processes including organismal development, viral infection, and cellular defense, thus causing a variety of human diseases (Klattenhoff et al., 2013; Ule et al., 2003). Several RBPs are associated with neurological disorders and cancer. For instance, Nova proteins are target antigens in the autoimmune disorder paraneoplastic opsoclonus–myoclonus ataxia (POMA), which gives rise to a neurodegenerative syndrome (Musunuru & Darnell, 2001). FMRP is an RBP highly expressed in brain. Absence or mutation of FMRP leads to the Fragile X syndrome, the most frequent cause of inherited mental retardation (Zalfa et al., 2003). Dementia and motor‐neuron diseases are associated with accumulation of disordered RBPs, such as TDP‐43 in amyotrophic lateral sclerosis (ALS) and ataxin‐1(ATXN1) in Ataxia (Bossy‐Wetzel et al., 2004). Also, perturbations in RBP‐RNA networks are causally related to cancer development. RBMS1 is ubiquitously expressed in triple negative breast cancer (TNBC). Researchers have found that RBMS1 regulates anti‐tumor T cell immunity in TNBC by regulating programmed death ligand 1 (PD‐L1) levels. As a new immune checkpoint regulator, RBMS1 has great potential to be the diagnostic marker and therapeutic target for TNBC (Zhang et al., 2022). The elucidation of RBP–RNA interaction is significant to understand underlying mechanisms in these biological and pathological processes, which has promoted the rapid development of methodology detecting RBP's target transcripts.

Currently, there are many methods for studying RBP–RNA interactions and they are mainly divided into two categories. The first category is RNA‐centric methods (Grawe et al., 2021; Ramanathan et al., 2019), which are designed to identify proteins that interact with a specific RNA of interest. This category includes both in vitro and in vivo methods. In vitro methods employ streptavidin‐binding biotinylated RNA (Zheng et al., 2016) or the S1 RNA aptamer (Leppek & Stoecklin, 2014), or use an engineered CRISPR endoribonuclease Csy4 (Lee et al., 2013) to immobilize bait RNA. A fluorescence‐labeled specific RNA can also be hybridized with a recombinant protein microarray chip to profile proteins binding to a given RNA (Kretz et al., 2013; Siprashvili et al., 2016). Among methods in vivo, chromatin isolation by RNA purification (ChIRP; Chu et al., 2011; Chu et al., 2015) and capture hybridization analysis of RNA targets (CHART; Simon et al., 2011; West et al., 2014) are based on a formaldehyde cross‐linking strategy, RNA antisense purification (RAP; McHugh & Guttman, 2018), peptide nucleic acid (PNA)‐assisted identification of RBPs (PAIR; Zeng et al., 2006), MS2 in vivo biotin tagged RNA affinity purification (MS2‐BioTRAP; Tsai et al., 2011), and tandem RNA isolation procedure (TRIP; Matia‐Gonzalez et al., 2017) rely on a UV cross‐linking strategy, while RNA–protein interaction detection (RaPID; Ramanathan et al., 2018) is a noncross‐linking method. The second category is protein‐centric methods, which focus on a specific protein of interest to identify its interacting RNAs. Cross‐linking and immunopurification (CLIP)‐based methods and RNA modification enzyme (RME)‐based methods are available in this category. Each method in the protein‐centric category has its unique advantages and limitations, making the choice of appropriate methods crucial for addressing specific biological questions.

## 2 THE DEVELOPMENT OF METHODS PROFILING RBPs TARGET RNAs

**FIGURE 1:**
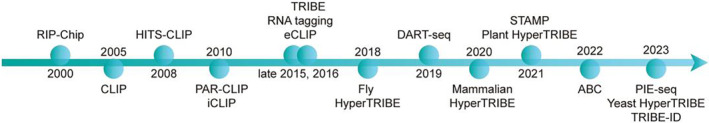
The timeline for development of RBP's target RNA profiling methods.

Over the last 25 years, a series of techniques with different accuracy and resolution have emerged for profiling RBP's target RNAs, and they can be roughly classified into CLIP‐based and RME‐based methods (Figure 1; Hafner et al., 2021; Lee & Ule, 2018; Ramanathan et al., 2019; Zarnegar et al., 2016). In 2000, Tenenbaum and colleagues developed RNA immunoprecipitation‐microarray (RIP‐Chip; Tenenbaum et al., 2000), and successfully profiled the mRNA compositions in the messenger ribonucleoprotein complexes (mRNPs) at a transcriptomic level for the first time. The method has been widely used, however, it has very obvious weaknesses like low target specificity and absence of binding site information, leading to difficulty in discovering RBP‐binding motif (Dahm et al., 2012). To overcome the weaknesses, several improved methods were generated. Ule and Darnell pioneered the ultraviolet (UV) crosslinking and immunoprecipitation (CLIP)‐based methodology. In 2003, they established an in vivo method CLIP (Ule et al., 2003, 2005), which makes covalent protein–RNA‐linking by irradiation of cells with UV and then purifies a specific protein–RNA complex using immunoprecipitation (IP) followed by SDS–PAGE separation. CLIP revealed dozens of mRNAs targeted and regulated by Nova protein in the brain, laying a foundation for the later exploration of the network of protein and RNA. In 2008, to accurately illustrate the RNA‐binding sites of an any given RBP on a large scale, Licatalosi and colleagues introduced the next‐generation sequencing (NGS) technology to CLIP method, and invented high‐throughput sequencing of CLIP cDNA library (HITS‐CLIP; Licatalosi et al., 2008). In 2010, the use of nucleotide analogs 4‐thiouridine (4‐SU) and 6‐thioguanosine (6‐SG) further improved the resolution and UV‐crosslinking efficiency of CLIP, and launched photocatalytivable ribonucleoside enhanced crosslinking and immunoprecipitation (PAR‐CLIP; Hafner, Landthaler, Burger, Khorshid, Hausser, Berninger, Rothballer, Ascano, et al., 2010; Hafner, Landthaler, Burger, Khorshid, Hausser, Berninger, Rothballer, Ascano, et al., 2010). In the same year, König and colleagues ingeniously utilized the characteristics that UV‐crosslinked RNA sites frequently induce mutations during reverse transcription (RT), and thereby developed individual‐nucleotide resolution CLIP (iCLIP; Konig et al., 2010, 2011), achieving nucleotide resolution in binding site identification.

Even though CLIP‐based methods had been optimized for several years, previously developed CLIP methods still had problems like high experimental failure rate, low reproducibility, high false‐positive signals, and low complexity of generated CLIP‐seq libraries. Thus, in 2016, Gene W. Yeo group developed the method enhanced CLIP (eCLIP; van Nostrand et al., 2016) based on the theory that reverse transcription is usually terminated at UV‐crosslinked RNA sites. eCLIP further improved the library preparation by ligating two linear adapters separately. As the second adapter was ligated to cDNA fragments at right after RT‐terminated 3′ ends, eCLIP could maintain the single nucleotide resolution in RBP‐binding site identification. Additionally, efficient enzymatic reactions, parallel IgG and size‐matched input controls, and using adaptors with unique molecular identifiers (UMIs) not only greatly increased library complexity and PCR duplicate identification accuracy, but also reduced background noise and adaptor ligation bias, resulting in more reliable results. Afterward, eCLIP was used to draw the large‐scale RBP–RNA maps and study the functions of 356 human RBPs systematically (van Nostrand, Freese, et al., 2020). To accomplish efficient large‐scale eCLIP experiments, Gene W. Yeo group developed a multiplexing eCLIP method, antibody‐barcode eCLIP (ABC), hiring antibodies with DNA barcoded in 2023 (Lorenz et al., 2023). In the ABC method, the antibody against a given RBP was linked with a barcoded adaptor, which is latter ligated to the RBP‐crosslinked RNA fragments after immunoprecipitation step. As immunoprecipitation of several RBPs could be conducted in a single tube, and the time‐consuming steps of SDS–PAGE running and membrane transfer were also removed in the ABC method, it is highly suitable for large‐scale experiments. The above advancements have ameliorated CLIP into a robust, multiplex, and scalable methodology for RBP target discovery.

Although CLIP and its derivative technology are powerful, they still have intrinsic features, such as depending on antibodies, requiring a large number of materials, UV crosslinking‐induced bias, and lacking isoform sensitivity. To develop methods with a totally different principle, scientists paid attention to a fusion protein system between RBPs and RNA modification enzymes (RMEs). As well known, RNA undergoes a variety of biochemical modifications, including methylation, 3′‐end tailing, and deamination (Boccaletto et al., 2022). In the RBP–RME fusion protein system, the RNA modification domain can make marks on RBP‐bound RNAs, and the marks can be identified by RNA sequencing. Wickens's lab and Rosbash's lab pioneered the RME‐based RBP's target RNA identification (Lapointe et al., 2015; McMahon et al., 2016).

In late 2015, RNA tagging method was developed in yeasts to reveal target RNAs bound by proteins, even like Bfr1p that do not have typical RNA‐binding domains (Lapointe et al., 2015). RNA tagging involves the fusion of a given RBP with the poly(U) polymerase (PUP) from *Caenorhabditis elegans*. Because PUP alone is not able to tether to mRNAs, it hardly uridylates mRNAs without assistance of other partners. While the RBP–PUP fusion protein can specifically add multiple uridines to 3′ terminals of target mRNAs which are bound by the RBP in the fusion protein. At the similar time, TRIBE was invented in flies (McMahon et al., 2016), which expresses the fusion protein of a RBP and the catalytic domain of *Drosophila*‐originated RNA editing enzyme ADAR (dADARcd) in vivo. The resulting A‐to‐I (read as G) RNA base‐editing events reveal the RBP's targets. Then, HyperTRIBE was established in flies in 2018 by introducing hyperactive E488Q mutation to dADARcd (Xu et al., 2018). HyperTRIBE was adapted in mammals in 2020 by employing catalytic domain of human ADAR2 (hADAR2cd) E488Q (Herzog et al., 2020; Jin et al., 2020). Later, HyperTRIBE was used in plants with dADARcd E488Q (Arribas‐Hernandez, Rennie, Koster, et al., 2021; Arribas‐Hernandez, Rennie, Schon, et al., 2021; Zhou et al., 2021) and in yeasts with hADAR2cd E488Q (Piao et al., 2023). In 2019, Meyer developed deamination adjacent to RNA modification targets (DART‐Seq) that can detect global m^6^A modification without using antibodies (Meyer, 2019). In 2021, Brannan et al. applied *Rattus norvegicus*‐derived C‐to‐U RNA editing enzyme rAPOBEC1 to development of surveying targets by APOBEC‐mediated profiling (STAMP) in mammals (Brannan et al., 2021). The tactics mentioned above will shed light on RBP's interactome in various organisms (van Nostrand, Pratt, et al., 2020).

Here, we focus on the RME‐based methods and introduce the RNA modification enzymes that are used in developing these methods. Also, we review the basic principles, advantages and disadvantages, and applications of the representative techniques, TRIBE, RNA tagging, STAMP, and their derivative methods.

## 3 ADENOSINE DEAMINASES THAT ACT ON RNA (ADAR) AND ITS DERIVATIVE TRIBE

### 3.1 The A‐to‐I RNA deaminase ADAR family

The A‐to‐I (G) editing is one of the most common RNA modification events in higher eukaryotes, and it is catalyzed by ADARs. ADAR family acts on double‐stranded RNA (dsRNA) regions and employs a base‐flipping mechanism to site‐specifically deaminate adenosines within duplex RNAs (Matthews et al., 2016). The mRNA editing can alter pre‐mRNA splicing, coded protein sequences, base pairing between mRNAs and miRNAs, circRNA biogenesis, and so on, thereby regulating gene expression and contributing to protein diversity (Beghini et al., 2000; Chen et al., 2013; Ivanov et al., 2015; Kawahara et al., 2007; Rosenthal & Seeburg, 2012; Wang et al., 2013). RNA editing can alter the structure and function of RNA, thus playing crucial roles in biological systems. Therefore, ADAR‐mediated RNA editing is associated with many human diseases (Chen et al., 2023; Nishikura, 2010; Slotkin & Nishikura, 2013; Song et al., 2022), including cancer (Chen et al., 2013), neurological disorders (Nakahama et al., 2021; Yang et al., 2021), metabolic disorders (Knebel et al., 2024), and immune diseases (Cai et al., 2023; Quin et al., 2021).

Most species have more than one *ADAR* homolog, however, ADAR family is not found in several nonmetazoan eukaryotes, such as yeasts, fungi, and plants (Savva et al., 2012). *ADAR1* and *ADAR2* are universally expressed in almost all tissues in mammals, whereas the expression of mammalian *ADAR3*, *Drosophila ADAR*, and *C*. *elegans ADAR1* are restricted mainly to nervous systems (Chen et al., 2000; Melcher et al., 1996; Palladino et al., 2000; Tonkin et al., 2002). Three members of ADAR family have been identified in human: *hADAR1*, *hADAR2* (Hajji et al., 2022), and *hADAR3*(Wang et al., 2017).

**FIGURE 2:**
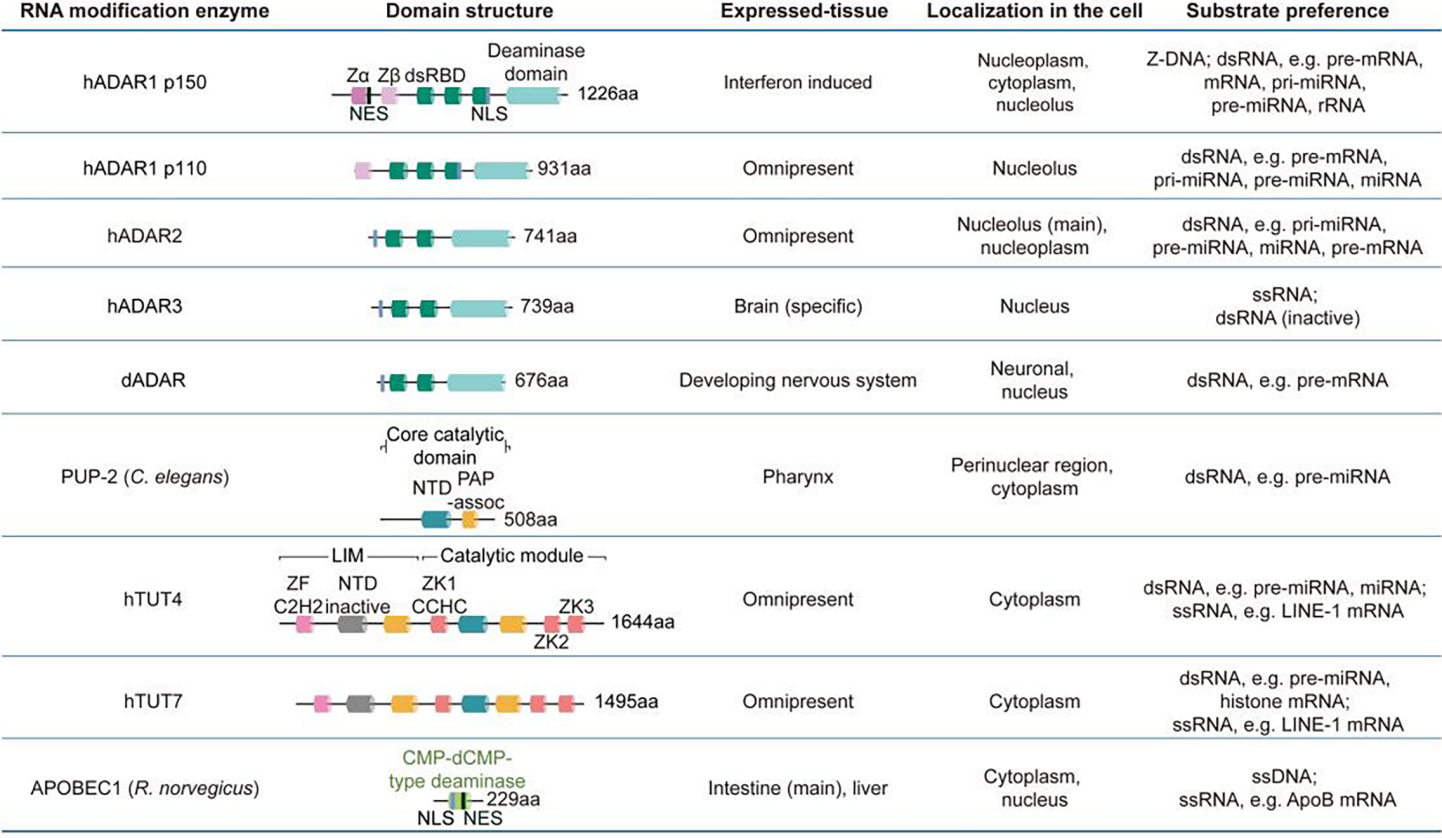
The characteristics of RNA modification enzymes. The main orthologues of ADAR, TENT, and AID/APOBEC family are listed. The same type of domains in uniform colors are designated for once in the figure. dsRBD, double‐strand RNA binding domain; LIM, Lin28‐interacting module; NES, nuclear export signal; NLS, nuclear localization signal; NTD, nucleotidyl transferase domain; PAP‐assoc, PAP‐associated domain; ZF C2H2, C2H2‐type zinc finger domain; ZK, CCHC‐type zinc knuckle domain; ZK1 CCHC, CCHC‐type zinc knuckle domain 1.

The *hADAR1* gene produces abundant protein isoforms of ADAR1 in human, hADAR1 p80, p110, and p150. ADAR‐1p80 localizes in the nucleolus in mouse and human while little is known about its editing capability (Figure 2; Lu et al., 2023; Yang et al., 2003). The short constitutively expressed isoform ADAR1–p110 is generated from multiple promoters, while the large protein isoform ADAR1–p150 can be induced by interferon through acting on the upstream interferon‐inducible promoter of *ADAR1* (Figure 2; George & Samuel, 1999; Patterson & Samuel, 1995; Xing et al., 2023). The ADAR1–p150 is believed to be involved in cellular defense against viruses and other pathogens (Sarkis et al., 2018). ADAR1–p110 is located in the nucleus due to the absence of nuclear export signal (NES), and p150 (Hayashi & Suzuki, 2013) is mainly localized in the cytoplasm (Poulsen et al., 2001). Furthermore, *mADAR1* has a conceptually similar gene organization and expression profile as the *hADAR1* (George et al., 2005).

The human *ADAR2* produces four protein isoforms of hADAR2a, 2b, 2c, and 2d with slight differences in C‐terminal deaminase regions, and hADAR2a and 2b are the major isoforms among them (Hajji et al., 2022; Lai et al., 1997). In vitro editing assay using GluR‐B transcripts as substrates has shown that hADAR2a and 2b have higher editing activity than hADAR2c and 2d (Lai et al., 1997).

The hADAR2 is localized to the nucleus, which is mediated by its N‐terminal nuclear localization sequence (NLS; Figure 2). Also, Pin1 binds to the phosphorylated Ser/Thr‐Pro motif in hADAR2 to isomerize the proline at 33 aa, assisting hADAR2 nuclear import (Marcucci et al., 2011). In *Pin1* knockout mouse fibroblasts, mislocalized hADAR2 is poly‐ubiquitinated and degraded in the cytoplasm by E3 ubiquitin ligase WWP2 mainly through Pro‐Pro‐x‐Tyr (PPxY) motif in both N‐terminus and C‐terminus of hADAR2 (Deffit & Hundley, 2016; Marcucci et al., 2011). So, the N‐terminus is important for hADAR2 nuclear localization and responsible for its instability in the cytoplasm. In addition, the change in subcellular localization of ADAR2 protein between the nucleoplasm and the nucleolus may serve as a mechanism to regulate endogenous ADAR2 substrate editing (Desterro et al., 2003; Sansam et al., 2003).

The *hADAR3* was supposed to originate from the duplication of the *hADAR2* gene, given the similarity in sequence and domain organization between ADAR2p and ADAR3p (Barraud & Allain, 2012; Chen et al., 2000). The ADAR3p is highly expressed in the brain, retains the ability to interact with dsRNA and ssRNA (single‐stranded RNA) but does not bring about any editing (Figure 2; Chen et al., 2000). Nonetheless, *mADAR3* was illuminated to have function in memory and learning in mice (Mladenova et al., 2018).

### 3.2 The substrate binding and editing specificity of ADAR

**FIGURE 3:**
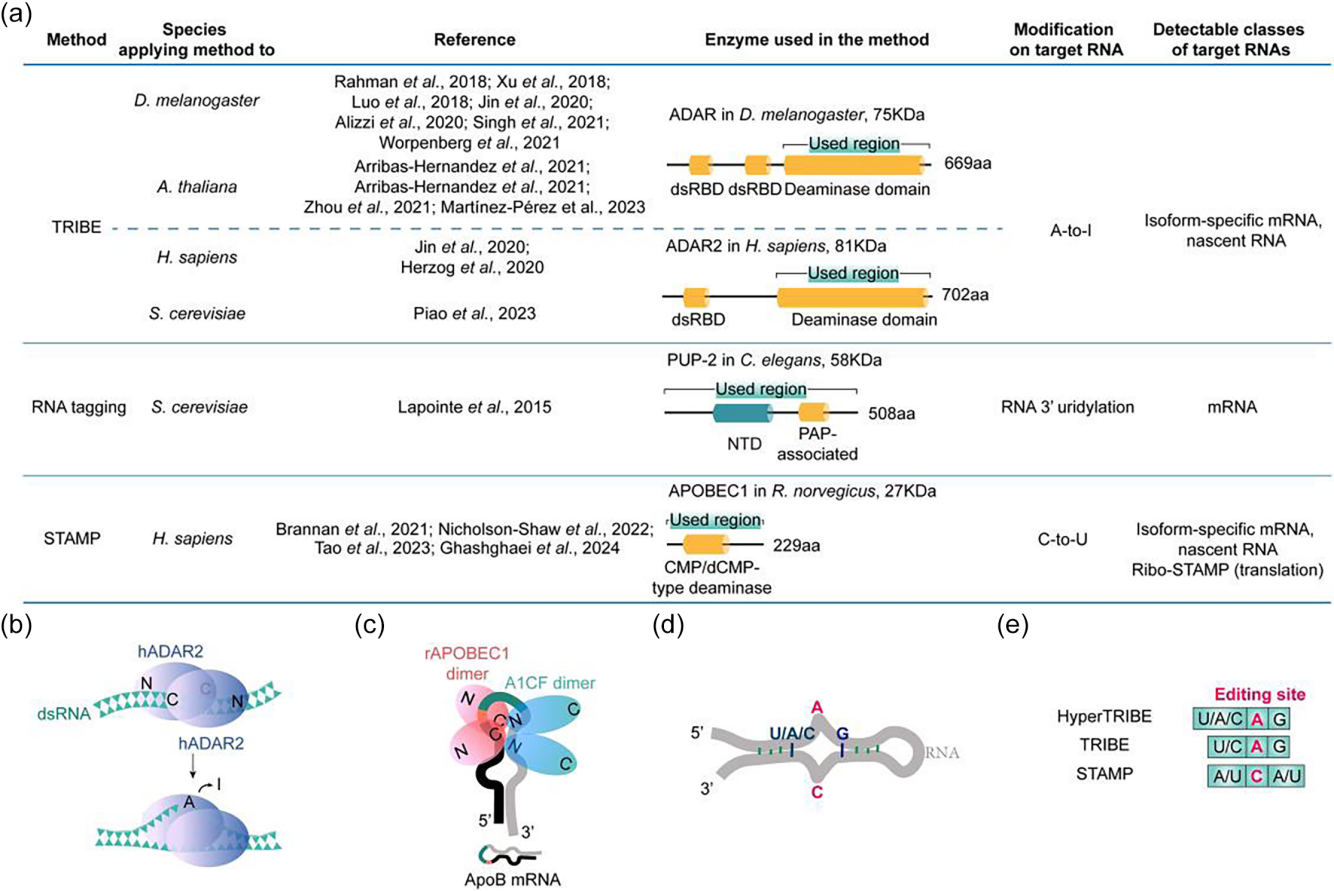
The detail of RME‐based RBP's target RNA profiling methods. (a) TRIBE, RNA tagging, and STAMP can determine different classes of target RNA by blending RBP with a special RNA modification enzyme (RME), comprising CePUP‐2, rAPOBEC1, or the catalytic domain of ADAR (ADARcd). (b) The model that hADAR2 binds to target under natural situations. The homodimer of hADAR2 binds to dsRNA, then local dsRNA structure is distorted, facilitating base‐flipping and deamination. (c) The model that rAPOBEC1 binds to ApoB mRNA under natural situations. A1CF homodimer (blue) together with rAPOBEC1 homodimer (pink) edit ApoB mRNA in the nucleus. ApoB mRNA includes regulatory sequence (black line), edited C (orange line), spacer sequence (blue line) and mooring sequence (gray line). N represents N‐terminal, C represents C‐terminal. (d) The preferred secondary structure and sequence in TRIBE/hyperTRIBE editing. (e) The nearest‐neighbor sequence preference for editing in different methods is showed. HyperTRIBE (dADAR E488Q) shows lower sequence bias for editing than in TRIBE (dADAR).

ADAR‐editing adenosines are typically situated within complete or nearly‐complete duplex structures (Phelps et al., 2015), both dsRBD (double‐strand RNA binding domain) and deaminase domain engage in base grabbing and adjustment of these substrates (Figure 2 and 3a; Deffit & Hundley, 2016; Wang et al., 2017). The dsRBD specifically recognizes its binding sites by both shape and sequence of RNA (Stefl et al., 2006, 2010; Stephens et al., 2004). Deletion experiment proved the nonessential role of N‐terminus and dsRBD1 of ADAR2 in editing 15 bp RNA substrate (Macbeth et al., 2004). On top of that, ZBD (Z‐DNA binding domain) of ADAR1 is related to Z‐conformation duplexes (Herbert & Rich, 2001), while ADAR3 carries an N‐terminal arginine‐rich domain for picking up ssRNA (Chen et al., 2000).

Although these nucleic acid‐binding domains primarily deposit ADARs near editing sites, the binding and editing are separate events. In other words, the binding isn't always followed by efficient editing, which only happens when appropriate editing sites are near the binding sites. Different deaminase domains, for example from hADAR1 and hADAR2, have their own substrate preference for editing (Kallman et al., 2003; Wang et al., 2018). ADAR2‐specific RNA‐binding loop and enzyme catalytic core in deaminase domain are in charge of the nearest‐neighbor preference for editing (Matthews et al., 2016; Wang & Beal, 2016). Homodimerization of ADAR on its target dsRNA enhances editing efficiency (Figure 3b; Barraud & Allain, 2012; Jaikaran et al., 2002; Poulsen et al., 2006; Stefl et al., 2010; Valente & Nishikura, 2007).

ADARs exhibit a multitude of editing sites due to nonstrict sequence specificity of reactions. There are the rules of ADAR catalysis: (1) The deaminase domain first catalyzes flipping‐out of adenosine from dsRNA. So, imperfect duplex structure is enough for deamination and A–C mismatch is dominant compared to A–U pair at the editing position (Kallman et al., 2003; Levanon et al., 2004; Wong et al., 2001). (2) Adenosines favor a 5′ neighbor of U or C and a 3′ neighbor of G for editing (Bazak et al., 2014; St Laurent et al., 2013; Wheeler et al., 2015). (3) Varied secondary structures of RNA, like bulges, hairpins, loops, and stems, have plus or minus effects on editing (Lehmann & Bass, 1999), which partly arises from specific recognition patterns of dsRBD (Stefl et al., 2006). (4) RNA tertiary structure is one of the origins of substrate specificity (Enstero et al., 2009).

To date, many amino acid mutants of ADARs have been identified in vitro (Matthews et al., 2016; Stefl et al., 2006) and in vivo screening (Kuttan & Bass, 2012; Poulsen et al., 2006; Wang et al., 2018, 2019; Wang & Beal, 2016; Wang, Havel, & Beal, 2015). The hyper active mutant hADAR1 E1008Q (Wang, Havel, & Beal, 2015) and hADAR2 E488Q (Kuttan & Bass, 2012) took the top spot, even though their catalytic efficiency depends on sequence‐context. Further engineering of ADAR proteins and inquiry into the edit‐aiding partner proteins will contribute to understanding of base editors and progress of RBP–RME methodology (Abudayyeh et al., 2019; Schneider et al., 2014; Stroppel et al., 2021).

### 3.3 TRIBE and HyperTRIBE identify RBP target transcripts based on ADAR2

**FIGURE 4:**
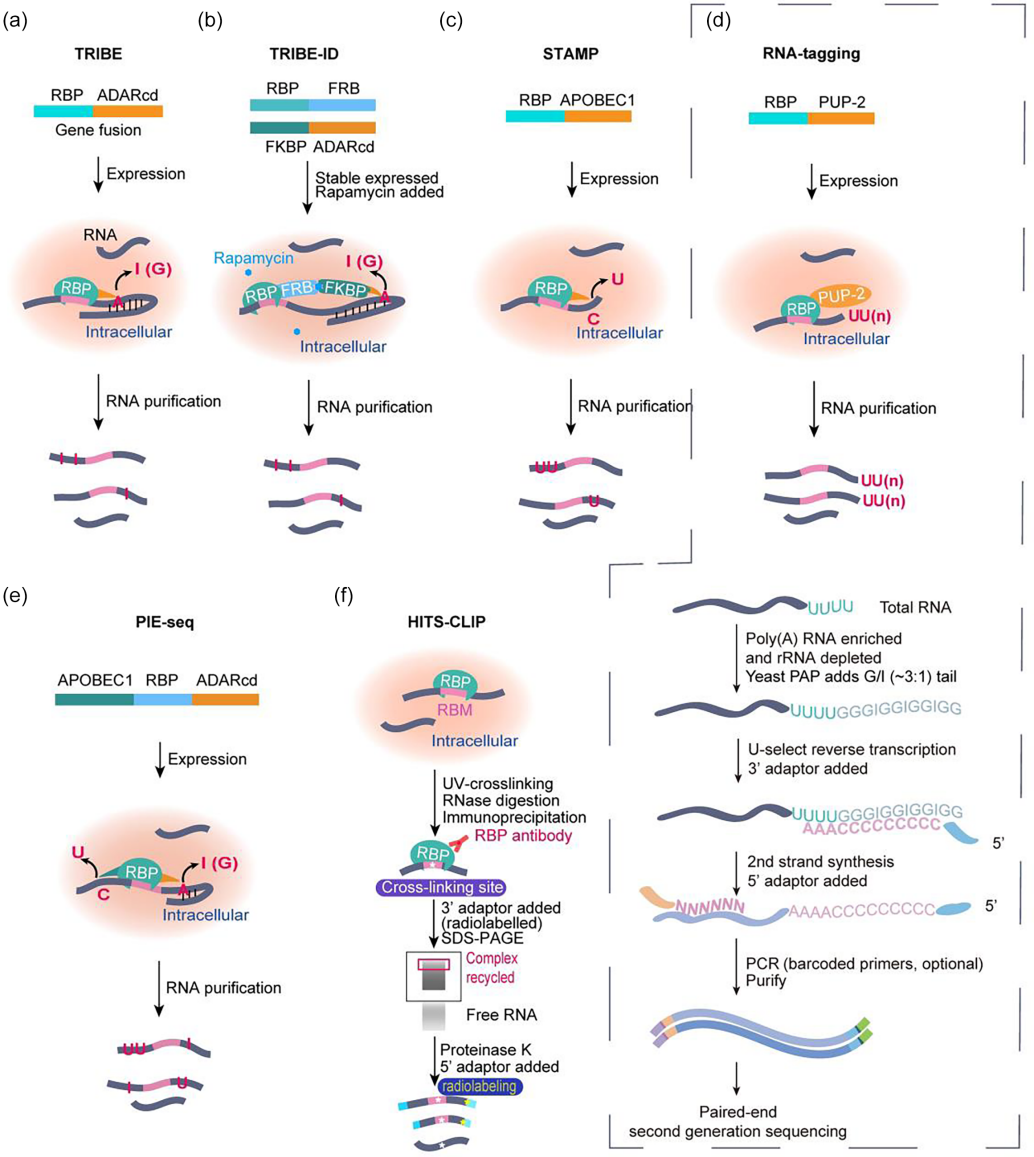
Schematic representation of TRIBE, TRIBE‐ID, STAMP, RNA tagging, PIE‐seq, and HITS‐CLIP. (a and b) TRIBE and its variant TRIBE‐ID can study RNA–protein interactions based on A‐to‐I (G) RNA editing. In TRIBE‐ID, the treatment of cells with rapamycin can induce FRP‐FKBP protein hetero‐dimerization inside cells, thus RBP–ADARcd complex is transiently formed and edits RBP's target RNA. (c) STAMP determines RNA targets with C‐to‐U edit. (d) RNA‐tagging expresses the fusion protein of RBP with tailing enzyme PUP‐2 (up) and employs a unique procedure of library preparation (down). (e) PIE‐seq expresses APOBEC1‐RBP‐ADARcd fusion protein and the target RNA molecules should have both A‐to‐I (G) and C‐to‐U edits. (f) HITS‐CLIP combines CLIP with high‐throughput sequencing for the first time. The same type of elements in the figure are lettered for once. ssDNA and ssRNA are arranged from 5′ to 3′ if there is no comment. ADARcd, ADAR catalytic domain.

TRIBE (McMahon et al., 2016) in flies entails the fusion of an RBP to the catalytic domain of *Drosophila* ADAR (RBP‐dADARcd), the fusion protein expressed in cells can put A‐to‐I editing tags on RBP‐bound mRNA in vivo (Figure 3a and 4a). Subsequently, these editing events can be pinpointed through high‐throughput mRNA sequencing and bioinformatics analysis. The samples of dADARcd‐only expression and no expression should be included in parallel so dADARcd‐mediated random editing and endogenous editing can be monitored and later be removed from targets sites. As mentioned above, N‐terminus of ADAR leads to its nuclear localization. In TRIBE, the N‐terminal dsRBD region of ADAR is substituted by RBP, so the localization of fusion protein depends on RBP. TRIBE makes 1–2 edits per target transcript on average, ~50% of edited sites are located within 100 nt from a CLIP peak, and ~80% are less than 500 nt from a CLIP peak (McMahon et al., 2016). Although TRIBE is not able to exactly map RBP‐binding positions, RBP‐binding motif can be identified by motif search with sequences around editing sites, for example with sequences ±100 nt of editing sites (McMahon et al., 2016). If the binding sites of a RBP are enriched in a specific region of mRNA, like 5′ or 3′ untranslated region (UTR), the sequences of enriched region from edited transcripts can be used for motif search (Jin et al., 2020). The motif search also needs to include a negative control composed of random sequences from non‐targets.

The RBP domain in TRIBE guides the fusion protein binding to RBP target mRNA, but TRIBE editing remains ADARcd‐derived strong preference for double‐stranded RNA region (Eggington et al., 2011; Macbeth et al., 2005; McMahon et al., 2016; Montiel‐Gonzalez et al., 2013; Phelps et al., 2015; Vogel et al., 2014; Vogel & Stafforst, 2014) especiclly a bulged A within dsRNA region (Eifler et al., 2013) even in the absence of ADAR dsRBDs (Figure 3d). In addition, the nearest‐neighbor of 5′ U/C and 3′ G are favored for editing (Figure 3d,e) while the exclusively single‐stranded regions and 5′ G are unsavory (Eggington et al., 2011; Kuttan & Bass, 2012; Porath et al., 2014).

Since TRIBE is limited by the low editing efficiency, as well as sequence and structural biases in editing, HyperTRIBE was developed by introducing hyperactive E488Q mutation into dADARcd (Rahman et al., 2018; Xu et al., 2018) in fly. The labeling efficiency improved more than 10‐fold in HyperTRIBE, sequence and structural biases were relieved, and false‐negative rate was dramatically cut. Subsequently, HyperTRIBE was adapted in mammals and yeasts with hADAR2cd‐E488Q (Herzog et al., 2020; Jin et al., 2020; Piao et al., 2023), in plants with dADARcd‐E488Q (Arribas‐Hernandez, Rennie, Koster, et al., 2021; Arribas‐Hernandez, Rennie, Schon, et al., 2021; Zhou et al., 2021).

In mammals, the catalytic domain from the protein isoform a of human ADAR2‐E488Q (hADAR2cd‐E488Q) was employed to make HyperTRIBE fusion protein (Herzog et al., 2020; Jin et al., 2020), beacuse the protein isoforms of hADAR2a and hADAR2b have more robust activity than those of hADAR2c and hADAR2d. Jin and colleagues employed HyperTRIBE to prove that eIF4E‐binding protein (4E‐BP) is associated with a specific set of mRNAs through eIF4E, under mTOR‐inhibited conditions in vivo in both *Drosophila* and mammals (Jin et al., 2020). Although some in vitro evidence had previously supported that 4E‐BP and eIF4E can bind to capped mRNA, 4E‐BP‐hyperTRIBE provided the first evidence confirming this association in vivo and explained how 4E‐BP preferentially inhibits translation of a subset of mRNA with TOP motif (Jin et al., 2020; Yang et al., 2022). Also, it is worth noting that TRIBE is a semi‐quantitative method and can be used to estimate relative protein–RNA interaction under different conditions (Jin et al., 2020).

With hyperTRIBE and other methods, Herzog and colleagues specified that TDP‐43 targets the mRNAs of several upstream regulators of CREB, thereby regulating CREB activity and dendritic branching. TDP‐43 dysfunction reduces transcription factor CREB activity and dendritic branching, so causes neurodegenerative diseases (Herzog et al., 2020).

Interestingly, MS2–TRIBE system could monitor spatial organization of transcription. In this system, MS2‐binding sites were inserted into an endogenous gene locus. During the transcription of the gene, ectopically‐expressed MCP (MS2 coat protein)‐ADARcd fusion protein can bind to the MS2 stem‐loop RNA region and make tags on spatially adjacent RNA, which gives the information about nuclear organization of transcription (Biswas et al., 2020). Anyway, even though hyper‐dADARcd can be used in mammalian HyperTRIBE (Biswas et al., 2020), its editing efficiency is weaker than hyper‐hADAR2cd in mammals (Jin et al., 2020). Thus, it is highly recommended to use hADAR2cd E488Q in mammalian HyperTRIBE.

In plants, several editing enzyme activities were compared for setting up HyperTRIBE, including adenosine deaminase (dADARcd E488Q and TadA of *E*.*coli* origin) and cytidine deaminase (rAPOBEC1, PmCDA1, and AtCDA1). Among them, the dADARcd‐E488Q performed well (Zhou et al., 2021) and was used to identify target mRNAs of a RBP AtUBP1c, and the m^6^A‐binding protein ECT2 and ECT3 in *Arabidopsis* (Arribas‐Hernandez, Rennie, Koster, et al., 2021; Arribas‐Hernandez, Rennie, Schon, et al., 2021; Martinez‐Perez et al., 2023; Zhou et al., 2021). In recent studies,the targets of Khd1p and Bfr1p were identified in *S*. *cerevisiae* by HyperTRIBE and yeast HyperTRIBE was successful with hADAR2cd‐E488Q (Piao et al., 2023).

The TRIBE approach facilitates the identification of cell‐specific (e.g., neurons) RBP–RNA interactions with minimal amounts of RNA (McMahon et al., 2016), which can be obtained by manual cell sorting, fluorescence‐activated cell sorting (FACS), or laser microdissection for plant tissues (Burjoski & Reddy, 2021). The transient expression of the fusion protein, for example, by inducible transcription system, is a better choice since long‐term expression will affect cell physiology too much and the edited targets are easily degraded by nonsense‐mediated mRNA decay (NMD) pathway. Another choice can be inducible protein dimerization between RBP and ADARcd like in TRIBE‐ID (Seo & Kleiner, 2023), which requires the expression of two fusion proteins, RBP–FRB (FKBP and rapamycin‐binding) and FKBP‐ADARcd (Figure 4b). FRB and FKBP interaction, or hetero‐dimerization, can be induced by rapamycin, so RBP's target RNA is only edited after adding rapamycin to cells in TRIBE‐ID. This method can avoid side effects from long‐term RBP‐ADARcd expression and quantify dynamic RNA–protein interactions in the form of inducible.

In summary, HyperTRIBE is a convenient, semi‐quantitative method, and is independent of antibodies. It has the advantage of examining RBP's targets in a small number of cells, being good to be used in the tissues with heterogenous cell types, as well as in organisms with low UV‐crosslinking efficiency because of cell wall and others. In addition, HyperTRIBE can determine indirect RBP–RNA interactions and be applicable in several representative organisms, including mammals, flies, and plants, by employing different origins of ADARcd‐E488Q.

## 4 TERMINAL NUCLEOTIDYL TRANSFERASE AND ITS DERIVATIVE RNA TAGGING

### 4.1 Terminal nucleotidyl transferase family

RNA tailing, nontemplated nucleotide addition in RNA 3′ termini, is a major type of mRNA modification (Doma & Parker, 2007; Martin & Keller, 2007; Munroe & Jacobson, 1990; West et al., 2006) and a common and conserved RNA processing pathway. Canonical poly(A) polymerase (PAP) adds poly(A) tails to the 3′ ends of mRNA during mRNA maturation in a transcription‐coupled manner in the nuclei of eukaryotic cells. Canonical PAP consists of an N‐terminal nucleotidyl transferase domain (NTD) and a C‐terminal RNA‐binding domain (RBD) with a nuclear localization signal (Liudkovska & Dziembowski, 2021). Terminal nucleotidyl transferases (TENTs) catalyze adenosylation, uridine acidification, guanylation, and cytidine acidification at the 3′ ends in the nucleus or cytoplasm (Norbury, 2013). TENT genes are well conserved in metazoan, including vertebrates, flies, and worms. Vertebrate TENT family can be sub‐classified into non‐canonical poly(A) polymerase (ncPAP) and terminal uridylyltransferase (TUTase) based on substrate preference for ATP or UTP. Some ncPAPs, like hTENT4A/4B, have broader substrate tolerance so generate A/G mixed‐tailing (Lim et al., 2018; Wen et al., 2023). It was suggested to refer to all vertebrate ncPAPs and TUTases by their respective TENT family names in the updated nomenclature and they typically include NTD and PAP‐associated domain (PAPd) but not RBD. The NTD and PAPd together comprise the core catalytic domain of TENT (Liudkovska & Dziembowski, 2021; Yu & Kim, 2020). In addition, CCA‐adding enzymes, poly(UG) polymerase (CeMUT‐2), and the enzyme with broad specificity (CeF31C3.2) were found in worms and fungi and all these RNA tailing enzymes other than canonical PAP were also called as noncanonical ribonucleotidyl transferases (rNTases; Preston et al., 2019).

### 4.2 Terminal uridylyltransferase subfamily

Terminal uridylyltransferases (TUTases) catalyze mono‐ and oligo‐uridylation of aberrant snRNA, miRNA, snoRNA, rRNA, 7SL, tRNA, Y and vault RNA, mRNA, and viral RNA in vivo, thereby playing key roles in their biogenesis, regulation, stability, and function (Liudkovska & Dziembowski, 2021; Warkocki et al., 2018; Yashiro & Tomita, 2018). They exist widely in mammals, plants, trypanosomes, worms, fruit flies, fission yeast, and other organisms. The hTUT1 (hTENT1) and hTUT4/7 (hTENT3A/3B) in human, Tailor (dTENT3) in fly, USIP1 (CeTENT1), and PUP‐1/2/3 (CeTENT3) in *C*. *elegans*, Cid1 and Cid16 in fission yeast have uridine acidification activity (Liudkovska & Dziembowski, 2021; Pisacane & Halic, 2017; Yu & Kim, 2020). Human TUT4/7 and CePUP‐1 (CDE‐1) are large multi‐domain enzymes, while the others, except for hTUT1, are simple and small proteins (Figure 2). PUP‐1/2/3 are essential for *C*. *elegans* germline development, PUP‐1 and PUP‐2 promote germline development redundantly under conditions of heat stress (Li & Maine, 2018). Similarly, TUT4/7 are important for gametogenesis and early embryonic development in mice (Morgan et al., 2017, 2019).

### 4.3 The processivity and substrate specificity of TUTases

The hTUT1 and its *C*. *elegans* ortholog USIP1 function in spliceosomal U6 snRNA biogenesis and regulation by shaping oligo(U) tails of U6. The hTUT1 carries RNA‐recognition motif (RRM), which possibly decides substrate specificity and gives enzyme processivity so binding to its substrates enough time for oligo‐uridylation (Liudkovska & Dziembowski, 2021; Ruegger et al., 2015; Trippe et al., 2006).

hTUT4/7 and its worm ortholog PUP‐2 (Poly(U) Polymerases‐2) participate in pre‐miRNA let‐7 degradation by Lin28‐dependent oligo‐uridylation of pre‐let‐7, making sure clearance of let‐7 in early animal development (Heo et al., 2009; Lehrbach et al., 2009). On the contrary, in the absence of Lin28 like in differentiated cells from late development stages, hTUT4/7 could only add mono(U) to pre‐let‐7 and promote the processing of pre‐let‐7 by Dicer (Heo et al., 2012). PUP‐2 possesses typical NTD and PAPd, but lacks any RNA‐binding domains, while in addition to NTD and PAPd, hTUT4/7 also consist of CCHC‐type Zink knuckle domain (ZK) and C2H2‐type Zink finger domain (ZF). The mono‐uridylation conformation requires NTD and PAPd of hTUT4/7 while oligo‐uridylation conformation needs not only NTD, PAPd, ZK2 of hTUT4/7 but also ZK of Lin28 (Figure 2; Faehnle et al., 2017). Thus, Lin28 is essential for hTUT4/7 and PUP2 processivity and oligo‐uridylation.

Interestingly, Tailor in fly oligo‐uridylates and degrades mirtron pre‐miRNA, which ends with 3′‐AG (Bortolamiol‐Becet et al., 2015; Reimao‐Pinto et al., 2015). The structural evidence indicates that R327 and N347 in Tailor core catalytic domain cooperatively contribute to substrate preference for 3′‐G and R327 possibly facilitates oligo‐uridylation (Cheng et al., 2019; Kroupova et al., 2019). So, in the case of Tailor, the core catalytic domain can support its processivity at least partly.

The mRNA and viral RNA are also the targets of TUTases in several organisms (Wen et al., 2023). The cellular mRNAs (Lim et al., 2014) and mouse hepatitis virus (MHV) genomic RNAs with short A‐tailed (<25 nt; Gupta et al., 2023), as well as the mRNAs of influenza A virus (IAV) are targeted by TUT4/7 for mono‐/oligo‐uridylation in mammals (Le Pen et al., 2018). The Orsay virus (OrV) RNA genome ends with mono‐U, CDE‐1 (PUP‐1) catalyzes mono‐uridylation of viral genome and resulted viral RNA with di‐U tails is degraded in OrV‐infected *C*. *elegans* (Le Pen et al., 2018). Some polyadenylated mRNAs are also uridylated by Cid1 and undergo mRNA decay pathway in *S*. *pombe* (Rissland & Norbury, 2009). Taken together, RNA oligo‐U‐tails trigger RNA degradations in many cases (Lubas et al., 2013; Malecki et al., 2013; Rissland & Norbury, 2009).

### 4.4 An assay TRAID‐seq for defining the TENT processivity and nucleotide preference in vivo

Apart from complicated endogenous mechanisms for substrate specificity, nucleotide preference, and enzyme processivity, a MS2‐based tethering and tailing assay TRAID‐seq (tethered rNTase activity identified by high‐throughput sequencing) performed in yeast can reveal the nucleotide preference and enzyme processivity own by enzyme‐self. In TRAID‐seq, the fusion protein between TENT and MS2 coat protein (MCP) was co‐expressed in yeast with the modified tRNA reporter bearing MS2‐binding sites (MBS) to specify TENT‐own biochemical activity in vivo (Preston et al., 2019). These can provide valuable information for TENT enzyme engineering and technology development, such as sequencing library preparation, molecular interaction detection, and hyper‐active enzyme screening.

In summary, mono‐ and oligo‐uridylation participate in biogenesis, decay, and function of coding and noncoding RNA. In many cases, oligo‐U tags often trigger spatiotemporally‐controlled RNA degradation, as well as abnormal RNA and viral RNA clearance, which plays key roles in embryonic development, cell cycle, circadian rhythm, immunity, and diseases (Beta & Balatsos, 2018; Chang et al., 2018; Le Pen et al., 2018; Liu et al., 2020; Reyes & Ross, 2016; Song et al., 2023).

### 4.5 RNA tagging identifies RBP target transcripts based on *CePUP* ‐*2* in yeast

A method called RNA tagging was developed, in which RBP was fused with terminal uridylyltransferase *CePUP*‐*2*. The coding sequences of *CePUP*‐*2* were inserted into downstream of a native RBP gene locus at *S*. *cerevisiae* genome thus the expression of fusion protein was under control of the endogenous RBP promoter (Figure 4d; Lapointe et al., 2015). CePUP‐2 carries only a minimal catalytic domain but not any RBD (Figure 2), and can add pretty pure U‐tails with high processivity in vivo when tethered to RNA in TRAID‐seq (Preston et al., 2019). RNA tagging employed CePUP‐2 to leave a covalent mark of U‐tails on RBP's target mRNAs and was applied to profile targets of two RBPs, Puf3 and Bfr1. About 50% sequencing reads in RNA tagging could be uniquely aligned to yeast genome, and hundreds to a thousand of targets were identified with U‐tag lengths ranging from 1 to more than 10 nt, meaning that these uridylated mRNAs were stable sufficient to be observed. These targets were also ranked according to U‐tag count and U‐tag length to quantify the affinity between RBP and target RNAs.

There are still some shortcomings in RNA tagging. At first, U‐tagging might be biased toward short A‐tailed mRNAs. It was reported that different organisms have varying poly(A) tail lengths, approximately 30 nt in yeast, 50 nt in *Arabidopsis* and *Drosophila*, and 70 nt in mammals on average according to PAL‐seq results (Subtelny et al., 2014). Also, adding oligo‐U to the end of poly(A) tail possibly induces target RNA degradation in some species (Lim et al., 2014). These will challenge adapting RNA tagging to other organisms. Second, RNA tagging does not provide information about the location of RBP binding sites, and has only been used in *S*. *cerevisiae* and not yet tried the RBPs, whose binding sites on mRNA far away from the ends of poly(A) tails to date.

Nevertheless, RNA tagging has avoided some complicated steps, such as cross‐linking, immunoprecipitation, radioactive‐labeling, gel‐running, turning to examination of uridylated RNA terminal. From the application aspect, RNA tagging can be applied for an individual type of cells or tissues, and for proteins directly and indirectly associated with mRNAs. This method is simple, reproducible, sensitive, and high‐throughput, exhibiting high performance in yeasts.

## 5 CYTIDINE DEAMINASE APOBEC AND ITS DERIVATIVE STAMP

### 5.1 The cytidine deaminase AID/APOBEC family

The AID/APOBEC polynucleotide cytidine deaminases can lead to C‐to‐T DNA mutation as well as Cytosine‐to‐Uracil (C‐to‐U) RNA editing. AID/APOBEC family includes activation‐induced cytidine deaminase (AID), ApoB mRNA editing enzyme catalytic‐1 (APOBEC1), APOBEC2, APOBEC3, and APOBEC4. Among them, APOBEC3 is specific to mammals, and is composed of a single gene in mice but expanded in primates into seven members, APOBEC3A (A3A), A3B, A3C, A3D, A3F, A3G, and A3H. APOBEC1 is conserved in tetrapods, AID and APOBEC2 are in vertebrates, whereas the orthologues of the mostly conserved APOBEC4 have even been found in invertebrates (Pecori et al., 2022).

According to substrate specificity, these members can be divided into two groups. The first group, C‐to‐U RNA editing enzymes APOBEC1, A3A, and A3G, can deaminate single‐stranded RNA (ssRNA). Peculiarly, they reserved the activity to deaminate single‐stranded DNA (ssDNA), which introduces mutations in genome and viral cDNA. As the first group enzymes can possibly deaminate both RNA and DNA, they were called as generalists. The second group, C‐to‐T DNA mutation enzymes AID and APOBEC2, can only catalyze the deamination of DNA but not RNA, so they are called as specialists. In fact, many members have not been categorized because of limited information about them (Pecori et al., 2022). Surprisingly, APOBEC2 was reported to act on DNA deamination‐independently (Powell et al., 2015). Humans carry the most diverse AID/APOBEC family members, including *AID*, *APOBEC1*, *APOBEC2*, seven *APOBEC3* genes, and *APOBEC4* (Salter et al., 2016). All members in this family are characterized by the catalytic domain of CMP‐dCMP‐type deaminase domain (Figure 2). Human A3B, A3D, A3F, and A3G have two deaminase domains, while the other members have only one. Some members additionally carry protein–protein interaction region and sub‐cellular localization signal, which are important for their functionality.

The loss of function studies has illustrated the importance of AID/APOBEC family roles. Mouse APOBEC1 functions in monocytic innate immune cells and nervous system by editing hundreds of target mRNAs (Cole et al., 2017; Rayon‐Estrada et al., 2017). APOBEC2 plays conserved roles in animal cardiac and skeletal muscle (Etard et al., 2010; Sato et al., 2010). APOBEC3 restricts viral infection and the transposition of genomic mobile elements through deaminating their cDNA intermediates (Bogerd et al., 2006; Harris & Dudley, 2015; Refsland & Harris, 2013). AID targets the genome regions of immunoglobulin genes in B cells thereby increasing antibody diversification (Muramatsu et al., 2000).

### 5.2 The substrate specificity of AID/APOBEC enzyme

*Rattus norvegicus* APOBEC1 (rAPOBEC1), the first member of AID/APOBEC family, was originally discovered in the ApoB mRNA editing event, in which rAPOBEC1 catalyzes C‐to‐U conversion on ssRNA substrates (Figure 2 and 3a; Navaratnam et al., 1993). The editing on ApoB pre‐mRNA generates a truncated protein isoform with distinct functions in lipid transport. Although APOBEC1 alone can catalyze the deamination of single cytosine in ApoB mRNA both in vivo and in vitro, the accessory protein APOBEC1 complementation factor (A1CF) and RNA‐binding motif protein‐47 (RBM47) are required for the efficient editing of ApoB mRNA (Blanc et al., 2019). The minimum required sequence for editing ApoB mRNA is 26 nt long (Figure 3c), which is highly conserved from marsupials to humans. Three cis‐acting elements are required for site specific deamination of ApoB mRNA (Backus & Smith, 1992; Smith et al., 2012; Soleymanjahi et al., 2021): an regulatory sequence upstream of editing C‐site (Figure 3c, black line), which regulates the editing efficiency (Maris et al., 2005); the flanking A/U at the 5′ and 3′ of the C‐site as well as an spacer in ~4 nt long (Figure 3c, blue line); the most important downstream 11 nt mooring sequence (Figure 3c, gray line), recognized by the accessory protein A1CF. In addition, extending the substrate's length to 102 nt enhances the editing efficiency (Wolfe et al., 2019).

Hundreds of transcripts are edited by APOBEC1 in mouse liver, intestine, and so on, and the ±4 nt AU‐rich flanking sequences are found adjacent to the APOBEC1 editing sites (Blanc et al., 2014; Rosenberg et al., 2011). The ~80% editing sites of APOBEC1 are located in 3′ UTR, some of the editing might affect the binding of miRNA to mRNA 3′ UTR so modulate the mRNA translation (Rayon‐Estrada et al., 2017). The substrate selection of APOBEC1 extremely depends on its co‐factor mainly RBM47 but also A1CF in mice, as illustrated by single or double knockout experiments of the co‐factors. RBM47 and A1CF specifically recognize mRNA, and directly recruit APOBEC1 protein for efficient mRNA editing. Each co‐factor has its own targets, they only share part of targets (Blanc et al., 2019; Pecori et al., 2022). Interestingly, the stem‐loop structure and cis‐acting elements similar to ApoB mRNA (Figure 3c) were also observed in these APOBEC1 substrates, and slight distinct features in the elements were expected to be recognized by different co‐factors (Soleymanjahi et al., 2021).

APOBEC1 is distributed in both the nucleus and the cytoplasm, but the nuclear distribution was observed only in cells that were capable of editing ApoB mRNA. It was reported that editing of ApoB mRNA almost occurred in the nucleus. The localization of APOBEC1 is regulated by both an N‐terminal nuclear localization signal (NLS) and a C‐terminal leucine‐rich nuclear export signal (NES), mutations on these regions impair substrate editing (Figure 2; Chester et al., 2003; Teng et al., 1999). Also, its localization may depend on the transport of accessory proteins, so C‐terminal hydrophobic domain of APOBEC1 related to intramolecular interactions further affects its subcellular localization (Wolfe et al., 2020).

The catalytic activity of AID/APOBEC family is provided by one or two conserved core zinc‐dependent cytidine deaminase domains. The catalytic domain comprises five β‐sheets (β1–β5) and is surrounded by six α‐helices (α1–α6), as well as 10 loops (L‐1 to L‐10) connecting them. The active site, a zinc finger motif H‐X‐E‐X_23‐28_‐P‐C‐X_2‐4_‐C (His‐Glu and Cys‐Cys, HECC), is highly conserved in the family (Revathidevi et al., 2021). The U‐shaped substrate binding groove on AID/APOBEC, defined by loops 1, 3, 5, and 7 surrounding the active site, interacts with U‐shaped 5–6 nt single‐stranded substrates and largely determines substrate specificity originated from enzyme itself. The amino acid variations in substrate binding groove might explain distinct preference for flanking sequence and DNA/RNA in different members of the family (Bohn et al., 2017; Kouno et al., 2017; Matsuoka et al., 2018; Rathore et al., 2013; Shaban et al., 2018; Shi et al., 2017; Wolfe et al., 2020). In addition, it was reported that the C‐terminal residues of rAPOBEC1 (196–210 aa and 221–229 aa) take part in protein homo‐dimerization (Teng et al., 1999), and C‐terminus‐deleted mAPOBEC1 (N1‐196 aa) neither dimerizes nor edits ApoB mRNA (Oka et al., 1997; Teng et al., 1999), RNA molecules are in turn required for dimerization of APOBEC1 (Ikeda et al., 2016).

### 5.3 STAMP identifies RBP target transcripts based on rAPOBEC1 in mammals

**FIGURE 5:**
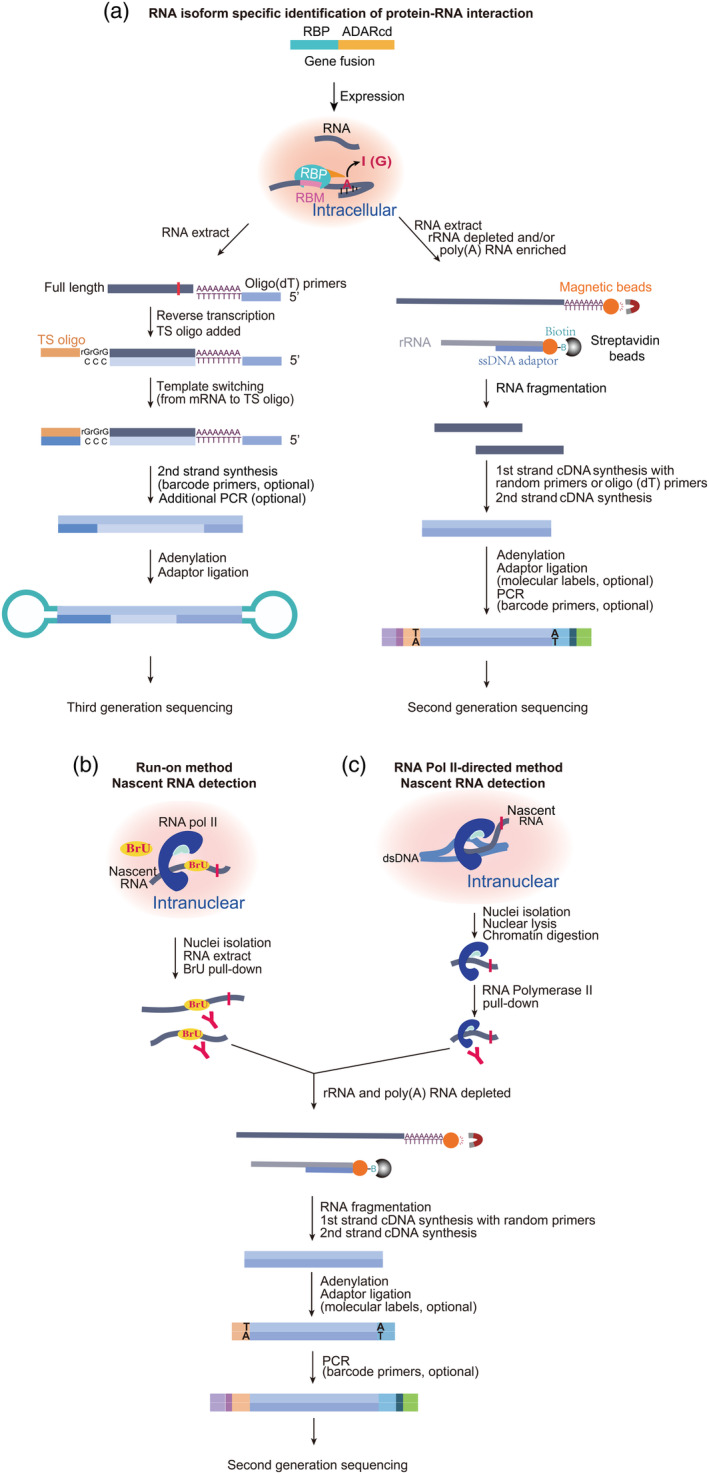
Schematic representation of methods for detecting isoform‐specific mRNA (TGS), poly(A) mRNA (SGS), and nascent RNA (Run‐on method and RNA pol‐II‐directed method). (a) The library preparation procedure for third or second‐generation sequencing (TGS or SGS) is simulated from the kits instruction of PACBIO® and illumina® respectively. Alternatively, rRNA can also be depleted through cleaving rRNA hybridized to DNA probes with RNaseH. (b and c) Run‐on and RNA pol‐II‐directed methods make no mutations on RNA and fit for nascent RNA target detection in RBP–RME method. Amalgamating with TRIBE is described as a model but it is also applicable to STAMP. The same kind of elements are presented for once in the figure, specific nucleotides are enlarged for convenience. BrU, Bromouridine.

DART‐seq (Deamination Adjacent to RNA Modification Target; Meyer, 2019) first mingled rAPOBEC1 with the m^6^A‐binding YTH domain to map m^6^A sites on mRNAs. Later, Gene W. Yeo group developed STAMP approach to demonstrate the RBP‐binding sites on RNA isoform‐specifically at single‐cell resolution (Figure 3a and 4c; Brannan et al., 2021). STAMP entirely follows the theory of immunopurification‐free identification of RBP targets by the fusion protein of full‐length rAPOBEC1 and a given RBP, but using extremely low or even single‐cell input. As RNA‐seq has thrived on deciphering RNA biology cell‐type specifically and isoform‐specifically with tools of spatial‐omics, single‐cell sequencing (scRNA‐seq), and long‐read RNA‐seq (Figure 5a; Stark et al., 2019), STAMP employed these tools. In a single pooled experiment, STAMP drew support from single‐cell capture and third‐generation long‐read sequencing methods and identified RNA–protein interactions in cell‐type‐specific and RNA isoform‐specific manners (Figure 5a). Also, the editing sites in RBFOX2‐STAMP were found within ±100 bp of expected RBFOX2 binding‐sites.

Furthermore, ribosome‐subunit STAMP (Ribo‐STAMP) bearing ribosomal subunit and rAPOBEC1 fusion offers ribosome‐interacting transcriptome (Brannan et al., 2021). The research tried two small ribosomal subunits RPS2 and RPS3, and RPS2‐STAMP exhibited better correlation when comparing EPKM (edited reads per kilobase of transcripts per million mapped reads) from Ribo‐STAMP with RPKM from ribosome‐protected fragments (RPFs) of Ribo‐seq. The editing sites in RPS2‐STAMP were mainly found in CDS but also in 3′ UTR, and 3′ UTR edits were confirmed to be effective in the quantification of translation efficiency and might partly arise from association small‐ribosomal subunit with mRNA even after translation termination. It was previously reported that the inhibition of mTOR pathway by its inhibitor like Torin‐1 represses translation preferentially for the mRNAs with 5’ TOP motif, which is enriched in the mRNAs of translational machinery, and further represses translation generally (Yang et al., 2022). RPS2‐STAMP could capture both general and specific translation repression after 72‐h treatment of mTOR inhibitor Torin‐1 (Brannan et al., 2021). While the requirement of time lag (12–24 h) to yield enough detectable edits dampens the capacity to monitor translational responses timely, and also nonsense or frameshift mutations cause artificial observation in Ribo‐STAMP. There is a demand for keeping expression levels of STAMP transgenes to an endogenous level to weaken false‐positive results.

The stem‐loop structure in APOBEC1 endogenous mRNA substrates is more likely needed for specific recognition by APOBEC1 co‐factors, the editing C‐sites are mainly positioned in ssRNA regions of the stem‐loops, such as loops, bulges, and tails (Soleymanjahi et al., 2021). The protein structures of core catalytic pockets of APOBEC1, A3A, and A3G indicate that they prefer binding to U‐shaped ssDNA/RNA (Kouno et al., 2017; Pecori et al., 2022). Therefore, STAMP is expected to edit ssRNA regions and does not require dsRNA structure for editing, even though this point needs further investigation. Anyway, the cytosine editing in STAMP still reserved the preference for 5′ and 3′ A/U rich flanking sequences (Figure 3e). The APOBEC1 alone and its co‐factor‐derived background edits can be removed by control STAMP expressing rAPOBEC1 only. When STAMP is applied in the nucleus, the possibility of genomic DNA editing should be considered. In addition, the subcellular localization of APOBEC1 is tightly regulated by its NLS, NES, and interacting co‐factors, therefore, it is necessary to check whether RBP‐rAPOBEC1 is properly localized prior to STAMP. STAMP has only been used in mammals to date (Brannan et al., 2021; Ghashghaei et al., 2024; Nicholson‐Shaw et al., 2022; Tao et al., 2023), and has shown unworkable in *Drosophila* (Abruzzi et al., 2023) and plant (Loeser et al., 2024), is still uncertain if it is applicable to other species.

### 5.4 Combined tribe with stamp

The principles of TRIBE (McMahon et al., 2016) and STAMP (Brannan et al., 2021) methods are similar, they fuse RBP with two different types of deaminase domain. TRIBE performs A‐to‐I (G) edits, and STAMP carries out C‐to‐U edits. Given the similarity in their underlying principles, it is feasible to combine these two methods for using together.

Abruzzi and colleagues compared hyperTRIBE and STAMP in both mammals and flies (Abruzzi et al., 2023). In human cells, both HyperTRIBE (TDP‐43‐hADAR2cd E488Q) and STAMP (TDP‐43‐rAPOBEC1) worked well with comparable performance regarding the numbers of identified editing sites per million reads and editing site reproducibility, and 70% of STAMP target genes were also observed in hyperTRIBE. Average editing percentage was higher in hyperTRIBE and mean number of editing sites per transcript was higher in STAMP (Abruzzi et al., 2023). The flanking sequence preference for editing in two methods was different as reported (Figure 3e). The cross‐checking with two methods would be more successful in identifying high‐confidence targets. Two methods (RBP‐dADARcd E488Q vs. RBP‐rAPOBEC1) were also compared in *Drosophila* cells, HyperTRIBE worked well as known but STAMP produced only a few editing sites, a similar level to the control editing. The same phenomenon was observed in plant (Loeser et al., 2024). As TRIBE and STAMP can study synergy and/or antagonism of two RBPs, it should put effort into adjusting STAMP in the species other than manmals.

Recently, PIE‐Seq was proposed (Ruan et al., 2023). It is a method of enhancing target fidelity by using double deaminases (Figure 4e). This method integrates an RBP into both hADAR2cd and rAPOBEC1. The PIE‐seq successfully identified target RNAs and binding motifs of 25 human RBPs, and was further applied to mouse brain for cell‐type specific profiling RBP–RNA interaction by single‐cell sequencing. PIE‐Seq reduces biases from enzyme preference for editing sequence context and secondary structure. Compared to a single deaminase, the dual deaminases of PIE‐seq enhance the fidelity of targets.

The TRIBE‐STAMP method (Flamand et al., 2022) applies TRIBE and STAMP simultaneously to two RBPs and observes whether two RBPs work on a single RNA molecule and how they do it (Owens & Liu, 2022). This method involves fusing one RBP with hADAR2cd‐E488Q (RBP1‐hyperTRIBE) and the other RBP with rAPOBEC1 (RBP2‐STAMP) and co‐expresses two constructs in mammalian cells. The authors applied TRIBE‐STAMP to the cytoplasmic m^6^A readers YTHDF1 (DF1), DF2, and DF3 to answer a long‐standing argument in the field (Flamand et al., 2022): One model had proposed that three paralogs have distinct pools of target genes and control their target RNAs in different ways; another model had suggested that DF1, DF2, and DF3 have redundant function in driving mRNA decay (Flamand et al., 2023). TRIBE‐STAMP was conducted with several combinations of m^6^A readers, and revealed that DF1, DF2, and DF3 share many target mRNAs and some target molecules could be bound by more than one DF paralogs throughout their lifetime (Flamand et al., 2022). They also observed that DF1 has more unique targets than the others and proposed the model: DF1 binding might not trigger rapid decay of mRNA molecules bound to it, so these mRNAs have chance to bind by DF2 or DF3 later; the mRNA molecules bound by DF2 might undergo rapid degradation so it has less unique targets.

Overall, combining TRIBE and STAMP could enhance detection accuracy, and be feasible to simultaneously study multiple co‐working RBPs regardless of the same complex or functioning redundantly, which has a wide range of applicability.

### 5.5 SCREENING RNA BASE‐EDITORS FOR DETECTION OF RBP–RNA INTERACTION

With the development of methods based on editing enzymes for detecting RBP–RNA interactions, it is particularly important to understand varying editing enzyme characteristics more thoroughly for application. The experimental and computational framework of PRINTER (Protein–RNA Interaction‐based triaging of enzymes that edit RNA; Medina‐Munoz et al., 2024) was designed in mammalian cells to evaluate the editing efficiency and specificity based on a MS2 stem loop‐MCP tethering reporter system. They screened 31 RNA base‐editors (rBEs) with A‐to‐I and/or C‐to‐U editing activity, and successfully identified 7 rBEs with expanded editing ability. These editors include C‐to‐U editors evoAPOBEC1 and APOBEC3A (Y132G/K30R), A‐to‐I editors TadA‐8e, TadA‐7.10, TadA‐7.10 (V82G), A‐to‐I and C‐to‐U dual editors ADAR2dd (R) and ADAR2dd (R‐S). Among them, APOBEC3A (Y132G/K30R) and TadA‐7.10 (V82G) exhibited the greatest editing activity and signal‐to‐noise ratio in the C‐to‐U and A‐to‐I rBEs, respectively. To identify two RBP targets concurrently, the APOBEC1 (C‐to‐U) and TadA 7.10 (V82G) (A‐to‐I) pair was recommended for tagging two RBP targets respectively in the same cells (Medina‐Munoz et al., 2024). In PRINTER, the activity of *Drosophila* ADAR E488Q (hyper‐dADAR/dADAR‐E488Q) was also tested and it showed low editing activity in mammalian cells. This result is consistent with the previous report that hyperTRIBE conducted with dADARcd‐E488Q in mammalian cells has low signal‐to‐noise ratio (Jin et al., 2020). Actually, hyperTRIBE in mammalian systems should carry out using catalytic domain of human ADAR2 E488Q (hADAR2cd‐E488Q) to achieve high signal‐to‐noise ratio (Jin et al., 2020). Although PRINTER was only conducted in mammalian system, the provided information will be valuable for adapting TRIBE and STAMP methods in varying species.

When applying rBE‐dependent detection methods, it is necessary to consider factors such as the preference of editing enzymes for flanking sequence and structure, and editing accuracy. In addition, it should be noted that higher enzyme activity may capture more RBP targets but also introduce higher noise, the optimal signal‐to‐noise ratio should be achieved as much as possible.

## 6 CONCLUSIONS

Despite the utility and strength of staple methodologies of RIP and CLIP, there are limitations in materials and conditions required, antibodies and a bulk of RNA quarried, native conditions of RIP raising nonphysiological interactions in vitro (Hafner, Landthaler, Burger, Khorshid, Hausser, Berninger, Rothballer, Ascano, et al., 2010; Konig et al., 2010; Licatalosi et al., 2008), and UV‐crosslinking desired to be enhanced (Mili & Steitz, 2004; Riley et al., 2012; Zhao et al., 2010), especially for RBP–dsRNA interaction mapping (Chi et al., 2009; Ricci et al., 2014). After overcoming all difficulties, fragmentation strategy of CLIP hides transcript isoform information and the detection of transient interaction in CLIP confounds false positives with biologically meaningful interaction (Figure 4f; Konig et al., 2010). Recent studies have made efforts to create fundamentally different methodology utilizing fusion of RNA modification enzyme to RBP of interest (RBP–RME), named TRIBE, RNA tagging, and STAMP (Jin et al., 2020; Lapointe et al., 2015; McMahon et al., 2016; Medina‐Munoz et al., 2020; Nguyen et al., 2020). In addition, Kleiner team developed RNA‐mediated activity‐based protein profiling (RNABPP; Dai et al., 2021, 2023), which is a metabolic labeling strategy based on reactive modified nucleoside probes to profile RNA modification‐associated proteins in living cells. This kind of approach will widen our knowledge about RME and further advance RBP–RME methodology.

To design an RBP–RME method, several things should be kept in mind. First, available RNA modification enzymes are varying according to organisms: dADARcd E488Q in fly and plant, hADAR2cd E488Q and CePUP‐2 in yeast, hADAR2cd E488Q, rAPOBEC1, APOBEC3A (Y132G/K30R), TadA‐7.10 (V82G), and others in mammals (Figure 3a). Second, the protein localization of RBP–RME should resemble natural RBP localization, not RME. It needs to avoid RME‐mediated mislocalization of RBP–RME. Third, transient and/or endogenous level expression of RBP–RME is recommended for reducing artificial observation. Fourth, when simultaneously exploring several RBPs, whatever they coordinate in one complex or function redundantly, a pair of RMEs are required like in TRIBE‐STAMP method (Medina‐Munoz et al., 2024): one with A‐to‐I editing and the other with C‐to‐U editing activities. The utilization of a TRIBE and STAMP pair can achieve target detection of two RBPs at a single molecular level. Fifth, Ribo‐STAMP advanced translation monitoring methodology. Since it is challenging to set up traditional translation exploration tools, like polysome profiling and Ribo‐seq, Ribo‐STAMP could be an option for the quantitative study of translation and applicable for the experiments of a single cell level (Einstein et al., 2021).

According to the purpose of the study and expected RBP‐binding regions in mRNAs, TRIBE and STAMP should choose different library preparation methods and high‐throughput sequencing. Regular downstream short‐read second‐generation sequencing (SGS) can identify targets at a gene level, long‐read third‐generation sequencing (TGS) can observe at an RNA‐isoform level (Figure 5a), and single‐cell sequencing can be used for cell‐specific monitoring of targets. If an RBP is expected binding to intronic regions of pre‐mRNAs, it should use the library preparation methods, which are able to investigate nascent RNAs. For example, it is possible to use global run‐on sequencing (GRO‐seq; Core et al., 2008), precision nuclear run‐on and sequencing (PRO‐seq) (up to single‐nucleotide resolution compared to the former) (Figure 5b; Kwak et al., 2013), RNA pol II‐directed method NET‐seq (aka native elongating transcript sequencing) (Figure 5c), or chromatin‐associated RNA (caRNA) methods. However, metabolic RNA labeling method is not appropriate because its U‐to‐C conversion will mix up with C‐to‐U edits left by APOBEC1 (Wissink et al., 2019). Many efforts have been made for establishing in yeast (Churchman & Weissman, 2011), fly (Prudencio et al., 2022), and mammalian (Nojima et al., 2015) NET‐seq or run‐on methods (O'Brien et al., 2023), for implementing caRNA in mouse (Menet et al., 2012) and fly (Khodor et al., 2011), which supplies convenience for amalgamating these methods with RBP‐RME methodology.

Since CLIP‐based and RBP–RME methods were designed with completely distinct principles, the methodologies in two different categories will favor revealing real RBP's target profile. Moreover, the following seven points need to be considered in order to choose a more suitable method among CLIP‐based and RBP–RME methods. First, RBP–RME methods can discern cell‐specific interaction between RBP and RNA, while CLIP‐seq cannot reach a single‐cell level. Second, only TRIBE and STAMP can do a survey for isoform‐specific interaction by using long‐read RNA‐seq, while RNA‐tagging is restricted by difficulty in PacBio sequencing library construction, and transcript digesting in CLIP‐seq will lose isoform details. Third, TRIBE and STAMP can detect nascent RNA targets while RNA‐tagging has not fitted for nascent RNA. CLIP‐seq can determine intronic RBP‐binding regions when combined with nuclei purification. Fourth, the complementary target mRNA tied by Argonaute (AGO)‐miRNA can be captured by CLIP (Chi et al., 2009; Rozen‐Gagnon et al., 2021), TRIBE (Sekar et al., 2023), and STAMP, but the object of small RNA (e.g., miRNA) can be only scanned by CLIP. It is believed that RBP–RME method is more effective in editing long RNA (about >100 nt). Fifth, TRIBE can acquire dsRNA targets efficiently while UV‐crosslinking between RBP and dsRNA in CLIP‐seq is likely inefficient. Sixth, CLIP‐seq can get binding data at nucleotide resolution, while TRIBE and STAMP cannot. Seventh, TRIBE‐STAMP can observe two RBPs working on a single RNA molecule (Flamand et al., 2022), whereas multiplexing eCLIP, the ABC method, can explore many RBPs concurrently (Lorenz et al., 2023).

RBPs are key regulatory factors of gene expression, and therefore their dysfunctions can lead to varying diseases. CRISPR‐Cas9 screening was carried out and identified dozens of RBPs, including YTHDF2, working on oncogenic pathways. With eCLIP, m^6^A sequencing, and single‐cell Ribo‐STAMP (scRibo‐STAMP), they further explored alterations in the translatome at a single‐cell level within YTHDF2‐depleted solid tumors and highlighted the therapeutic potential of YTHDF2 in breast cancers (Einstein et al., 2021). In addition, RNA‐binding protein Ataxin‐2 (Atx2) is associated with the pathogenesis of various neurodegenerative diseases. The extensive data set of Atx2‐target mRNAs were identified by TRIBE in *Drosophila* (Singh et al., 2021), which contributed to the knowledge of neural translation control mechanism and provided a therapeutic strategy targeting human Atx2. The critical proliferation‐related role of CCDC137 was demonstrated in hepatocellular carcinoma (HCC) patients by STAMP (Tao et al., 2023). Therefore, the continuous development and improvement of RBP–RNA interaction profiling technology will benefit the understanding of human health and disease mechanisms.

The RME‐based strategy has made up a substantial portion of mRNA target identification. Here, we reviewed RNA modification enzymes in ADAR, TENT, and AID/APOBEC family, regarding the biological function, biochemical activity, and substrate specificity of the enzymes and their domains. Also, some protein mutations with engineered activity were discussed. Furthermore, we provided a systematic overview of the basic principles, advantages, disadvantages, and applications of the RNA–protein interaction identification techniques, such as TRIBE, RNA tagging, STAMP, and CLIP‐seq. These distinctive methods will keep contributing to the study of RBP–RNA interactome.

## AUTHOR CONTRIBUTIONS

**Hua Jin:** Funding acquisition (lead); project administration (lead); supervision (equal); validation (equal); writing – review and editing (equal); writing – original draft (lead); . **Chong Li:** Validation (supporting); visualization (lead); writing – review and editing (equal). **Yunxiao Jia:** Visualization (supporting); writing – review and editing (supporting). **Weilan Piao:** Supervision (equal); validation (equal); writing – original draft (lead); writing – review and editing (equal). **Yuxuan Qi:** Validation (equal); writing – review and editing (equal).

## FUNDING INFORMATION

The work was supported by the General Program of the National Natural Science Foundation of China (31970622), and supported by the Fundamental Research Funds for the Central Universities.

## CONFLICT OF INTEREST STATEMENT

The authors have declared no conflicts of interest for this article.

## RELATED WIREs ARTICLE

To edit or not to edit: Regulation of ADAR editing specificity and efficiency

## Data Availability

Data sharing is not applicable to this article as no new data were created or analyzed in this study.
